# Migration Rate Inhibition of Breast Cancer Cells Treated by Caffeic Acid and Caffeic Acid Phenethyl Ester: An In Vitro Comparison Study

**DOI:** 10.3390/nu9101144

**Published:** 2017-10-19

**Authors:** Agata Kabała-Dzik, Anna Rzepecka-Stojko, Robert Kubina, Żaneta Jastrzębska-Stojko, Rafał Stojko, Robert Dariusz Wojtyczka, Jerzy Stojko

**Affiliations:** 1 Department of Pathology, School of Pharmacy with the Division of Laboratory Medicine in Sosnowiec, Medical University of Silesia in Katowice, Ostrogórska 30, 41-200 Sosnowiec, Poland; rkubina@sum.edu.pl; 2 Department of Pharmaceutical Chemistry, School of Pharmacy with the Division of Laboratory Medicine in Sosnowiec, Medical University of Silesia in Katowice, Jagiellońska 4, 41-200 Sosnowiec, Poland; annastojko@sum.edu.pl; 3 Department of Anesthesiology and Intensive Care, Prof. K. Gibiński University Clinical Center, Medical University of Silesia in Katowice, Ceglana 35, 40-514 Katowice, Poland; zak@czkstojko.pl; 4 Department of Women Health, School of Health Sciences, Medical University of Silesia in Katowice, Medyków 12, 40-752 Katowice, Poland; rstojko@sum.edu.pl; 5 Department and Institute of Microbiology and Virology, School of Pharmacy with the Division of Laboratory Medicine in Sosnowiec, Medical University of Silesia in Katowice, Jagiellońska 4, 41-200 Sosnowiec, Poland; rwojtyczka@sum.edu.pl; 6 Department of Toxicology and Bioanalysis, School of Pharmacy with the Division of Laboratory Medicine in Sosnowiec, Medical University of Silesia in Katowice, Jagiellońska 4, 41-200 Sosnowiec, Poland; jstojko@sum.edu.pl

**Keywords:** caffeic acid, CAPE, migration, wound healing, breast cancer, propolis

## Abstract

One of the deadliest cancers among women is a breast cancer. Research has shown that two natural substances occurring in propolis, caffeic acid (CA) and caffeic acid phenethyl ester (CAPE), have significant anticancer effects. The purpose of our in vitro study was to compare cytotoxic activity and migration rate inhibition using CA and CAPE (doses of 50 and 100 µm) against triple-negative, MDA-MB-231 breast adenocarcinoma line cells, drawn from Caucasian women. Viability was measured by XTT-NR-SRB assay (Tetrazolium hydroxide-Neutral Red-Sulforhodamine B) for 24 h and 48 h periods. Cell migration for wound healing assay was taken for 0 h, 8 h, 16 h, and 24 h periods. CAPE displayed more than two times higher cytotoxicity against MDA-MB-231 cells. IC_50_ values for the XTT assay were as follows: CA for 24 h and 48 h were 150.94 µM and 108.42 µM, respectively, while CAPE was 68.82 µM for 24 h and 55.79 µM for 48 h. For the NR assay: CA was 135.85 µM at 24 h and 103.23 µM at 48 h, while CAPE was 64.04 µM at 24 h and 53.25 µM at 48 h. For the SRB assay: CA at 24 h was 139.80 µM and at 48 h 103.98 µM, while CAPE was 66.86 µM at 24 h and 47.73 µM at 48 h. Both agents suspended the migration rate; however, CAPE displayed better activity. Notably, for the 100 µM CAPE dose, motility of the tested breast carcinoma cells was halted.

## 1. Introduction

Properly-fortified diets, especially those enriched with compounds such as polyphenols or phenolic acids, are found to counter the development of many diseases [1,2,3].

Propolis is one of many natural substances which are becoming increasingly popular for study by cancer research projects. Propolis is an amorphous, viscous substance, of a resin-like consistency, produced by honey bees (*Apis mellifera*) from collected plant pollen, which bees supplement with bee’s wax or bee bread. The composition of propolis is extremely complex and varies depending on the locale, season, weather conditions, and plant species from which it is gathered [4,5,6,7].

Numerous scientific studies have shown that propolis has antiviral, antimicrobial, antifungal, and antiparasitic properties, as well as being known to have anti-inflammatory and antioxidant effects. Additionally, active, cardio-, and hepatoprotective effects have been measured using propolis. There are also research reports of propolis being used as a local anesthetic. Some authors report this compound has antitumor properties due to the antiproliferative, cytotoxic, and proapoptotic properties of propolis compounds. The high concentration of these substances in propolis determine its anti-inflammatory, anti-microbial, regenerative, immunomodulatory, hepatoprotective, antioxidative, and therefore antitumor activity [4,5,6,7,8,9,10,11,12,13,14,15,16,17,18].

There are hundreds of substances occurring in propolis. Two of these are caffeic acid (CA) and the caffeic acid phenethyl ester (CAPE). Some of their properties reported in research include: antioxidative, antibacterial, antiviral, anti-inflammatory, antiplatelet, antitumor, and antineoplastic effects [19,20,21,22,23,24,25].

In vitro research studies have clearly shown the cytotoxic properties of CAPE against: cells of pulmonary carcinoma, gastric carcinoma, colorectal carcinoma, malignant melanoma, hepatic carcinoma, pancreatic carcinoma, as well as cervical carcinoma [26,27,28,29,30,31,32,33].

Our earlier research showed that CA inhibits the viability and migration process of oral carcinoma SCC-25 cells and head and neck squamous carcinoma cells, while CAPE was directly reported as a growth inhibitor of breast cancer cells [34,35].

CAPE’s antitumor activity was also reported. CAPE inhibits activity of cancer cells by using the significant nuclear transcription factor NF-κB. NF-κB inhibits apoptosis, induces proliferation, and intensifies angiogenesis. Evidence shows that NF-κB could be one of the most important factors in the process of oncogenesis and cancer progression. Moreover, it was shown that CAPE aggregates the Fas death-inducing receptors through a Fas-L-independent mechanism [36,37,38].

Research studies of breast cancer have shown CAPE is an inhibitor of FGF-2 (fibroblasts growth factor type 2), which is a factor of tumor growth. CAPE performance was evaluated positively, both for in vitro and in vivo research of MCF-7 and MDA-231 breast cancer cells. It is worth noting that CAPE did not affect healthy cells [23,39]. It was also shown that CAPE reduces expression of the mdr-1 gene, which causes increased sensitivity of tumor cells to chemotherapy. Also, after treatment with CAPE, the decreased vascular endothelial growth factor (VEGF) inhibited angiogenesis and tumor growth [40,41].

Breast cancer is one of the deadliest cancers among women. It is well known that genetic changes are conducive to the development of cancer. For instance, activation of oncogenes and alteration of tumor suppressor gene pathways leads to the development of tumor cells. Eventually, tumor cells lose complete control from highly regulated cell growth signals, leading to abnormal proliferation and avoiding apoptosis [42].

Breast cancer is a heterogeneous disease with many biological subtypes. One is triple negative breast cancer (TNBC). TNBC is much more aggressive than breast cancer of other molecular subtypes and known for its frequency of recurrence. As a result, the mortality rate of patients with TNBC is significantly higher than among patients with other types of breast cancer (estrogen receptor α and progesterone receptor positive) [43].

The treatment of a triple negative breast carcinoma depends on the severity of the disease. The choice of therapy is affected by the presence of metastases, the size of the primary tumor, and the result of detailed pathological tests, such as the degree of malignancy of the tumor, which determines the rate of division of tumor cells. Surgical methods, radiotherapy, and chemotherapy are used in the treatment of triple negative breast cancer. In the treatment of patients with TNBC, it is important to inhibit a formation of blood vessels providing nutrients to tumor tissues, the angiogenesis. However, the therapeutic options are very small here, but neither hormone therapy nor HER2 drugs works. Therefore, the average survival time in this group is still less than in patients with other types of breast cancer. The biological characterization of the tumor cell is the method used to determine the type of tumor and to obtain valuable information on the factors influencing its growth. Triple negative breast cancer is diagnosed in patients under the age of 50. Factors that promote TNBC are: early menopause, obesity at menopausal age, and breast cancer in the family. The disease is characterized by an aggressive course, rapid tumor growth, and rapid onset of distal metastases (especially to the brain and lungs, and to a lesser extent bone and liver) and early relapse (within 1–3 years of diagnosis). A large number of patients with this type of cancer have a poor prognosis due to low remission during adjuvant therapy, and in the case of metastases a short survival time and high resistance to chemotherapy [44,45,46,47,48].

The response rate among patients with metastatic breast cancer is gradually decreasing, possibly due to the tumor’s resistance to a wide range of cancer therapies [43,49,50,51]. Unfortunately, it must be mentioned that metastatic breast cancer’s resistance to all forms of systemic treatments (hormonal, chemotherapy, and targeted) results in an estimated 90% or more of patients with metastatic disease developing tumors which will prove lethal [52].

The aims of decreasing toxicity levels of standard breast cancer treatment and increasing patients’ survival chances have led researchers to test natural substances and their varying compounds. Such studies have yielded highly positive and encouraging results for breast cancer treatment. Taking into consideration the above facts, we compare in vitro effects of CAPE and CA (viability and migration) on MDA-MB-231 human breast cancer cells, which to the best of our knowledge is a new approach.

## 2. Materials and Methods

### 2.1. Cell Lines and Reagents

#### 2.1.1. Breast Cancer Cell Line MDA-MB-231

In this research, the MDA-MB-231 line (human breast adenocarcinoma TNBC, No. 92020424 SIGMA from Sigma-Aldrich, Poznań, Poland) was used, as it is a model of human triple-negative breast cancer. The manufacturer’s recommendations for preparations were all carefully followed. The MDA-MB-231 cells were cultured with Leibovitz’s L-15 medium, with 10% of inactivated fetal bovine serum (FBS, Sigma-Aldrich, Poznań, Poland), and kept at 37 °C, without CO_2_.

All cultured cells were supplemented with antibiotics of the following concentrations: penicillin—100 U·mL^−1^, streptomycin—100 μg·mL^−1^ and fungistatic amphotericin B with a concentration of 0.25 μg·mL^−1^. The medium was changed every 2–3 days, with the passage carried out with a confluence of 80% to 90%.

#### 2.1.2. CA and CAPE

Both caffeic acid (CA, Sigma: C0625) and caffeic acid phenethyl ester (CAPE, Sigma: C8221) were purchased from Sigma-Aldrich, Poznań, Poland and were collected, stored, and used specifically according to the manufacturer’s instructions.

### 2.2. Microscopic Evaluation of Carcinoma Cells Morphology. Hematoxylin and Eosin Staining Protocol

Initially, the MDA-MB-231 cells were inoculated into 2-chamber microscopic culture vessels (Lab-Tek, Waltham, MA, USA) at a count of 1000 cells/well. Depending on the time of the experiment, we added proper concentrations of the studied compounds to the media and left them for 24 and 48 h. This was followed by leaving the cultures for 24 h to obtain the cells’ growth rate. They were then fixed for 12 h in 96% ethanol. The cells were then hydrated in the following series of dilutions: 99.6%, 96%, 90%, 80%, 70%, and 50% and stained with hematoxylin for 7 min (standard H&E staining protocol). Next, the plates were washed with PBS solution for approximately 30 min to blue up and were then incubated for 30 s with eosin. PBS solution was used again to wash the plates, and they were then dehydrated with ethanol of increasing concentrations of 50%, 70%, 80%, 90%, 96%, and 99.6%. Finally, the plates were immersed in the ethanol and xylene mixture (50:50) for 1 min and then in pure xylene. The plates were then mounted and analyzed under a microscope.

### 2.3. Cell Viability by Mitochondrial Activity, XTT Test Assay

Viable cells depend on an intact mitochondrial respiratory chain and an intact mitochondrial membrane. Activity of the measured compounds was determined using mitochondrial dehydrogenases from the viable cells. XTT (2,3-bis[2methoxy-4-nitro-5-sulfopheny]-2H-tetrazolium-5-carboxyanilide inner salt) is a tetrazolium salt that cleaves to formazan by the succinate dehydrogenase system, which belongs to the mitochondrial respiratory chain. This is significant, as it is only active in viable cells. Mitochondrial succinate dehydrogenase reduces yellow tetrazolium salt into a soluble orange formazan in the presence of an electron coupling reagent. The number of originating formazan is proportional to the amount of living cells [53]. We measured the enzyme activity at 480 nm, which is in line with the manufacturer’s recommendation. The XTT assay was obtained from Xenometrix AG, Allschwil, Switzerland.

To measure cytotoxicity, the cells were inoculated on 96-well plates, at an amount of 10^4^ cells/well. A fresh medium was added and left for 72 h to obtain the rate of cell growth. After the medium was decanted, separate culture mediums were added which contained 50 µM and 100 µM of either CA or CAPE, which had been prepared during a series of dilutions in the culture medium. A measure of 0.1 mL of medium with the defined concentrations of the substances was added to each well and left for 24 h/48 h in a CO_2_ incubator at 37 °C. The test procedure was performed exactly in accordance with the instructions and protocol of the manufacturer.

### 2.4. Cytotoxicity by Lyzosomal Activity, NR Test Assay

Cell survival and viability can also be measured by using the ability of viable cells to incorporate and bind to neutral red (NR), which is best performed on adherent cells. Neutral red is a weak cationic dye that readily penetrates the cell membrane and accumulates intracellularly in lysosomes (lysosomal pH < cytoplasmic pH), where it binds to anionic sites of the lysosomal matrix. Lysosomal fragility and other effects, which gradually became irreversible, are caused by changes of the cell surface and the sensitivity of the lysosomal membrane. Such alterations, induced by the action of xenobiotics, result in decreased uptake and binding of NR. Therefore, it is possible to distinguish between viable, damaged, or dead cells [54]. The NR test was obtained from Xenometrix AG, Allschwil, Switzerland. We used CA and CAPE at concentrations of 50 µM and 100 µM, with 24 h and 48 h of incubation. The quantity of dye incorporated into cells was measured by spectrometry at 540 nm, which is directly proportional to the number of cells of the intact membrane. Test procedures were performed exactly following the instructions and protocol of the manufacturer.

### 2.5. Cell Proliferation by SRB Test Assay

Cell proliferation, measured as total protein synthesis, is a very sensitive toxicology marker. Sulforhodamine B (SRB, Acid Red 52) is an anionic dye that binds electrostatically to cellular proteins. SRB binds stoichiometrically to cellular proteins (when mild acidic conditions are guaranteed) and can then be extracted under basic conditions. The total amount of bound dye can be used as a proxy for cell mass, which is directly proportional to cell proliferation [55,56]. A fixed dye was solubilized and measured photometrically at OD 540 nm, with a reference filter of 690 nm. The OD values were correlated with total protein content and therefore with cell number. Concentrations of CA and CAPE of 50 µM and 100 µM were used to make the experiments for 24 h and 48 h of incubation. The SRB test was obtained from Xenometrix AG, Allschwil, Switzerland. Procedure of the test was performed exactly in accordance to the instructions and protocol of the manufacturer.

### 2.6. Migration—Cell Wound Closure Assay

Carcinoma cell migration is the result of variable biological processes, with a specific characteristic seen in their coordination. Wound-healing assays are standard and commonly used methods for investigation of cell migration. CA’s and CAPE’s ability to modify cell motility using the scratch wound healing assay was then analyzed [57,58]. This method was implemented to evaluate the migration activity rate of MDA-MB-231 cells exposed to CA and CAPE.

Briefly, the MDA-MB-231 cells (4 × 10^6^ cells/well) were plated in 6-well plates for 48 h to a confluence of about 80%, then wounded by scratching with a p200 pipette tip. Thereafter, the debris was removed and we washed the cells once with 1 mL of the growth medium to assure the edges of the scratch were smoothed by washing. We took utmost care to make the wounds of the same dimensions, both for the experimental and control cells to minimize any possible variety resulting from a difference in scratch width.

The cells were then incubated with DMEM medium containing 0.5% FBS and treated with CA/CAPE doses of 50 μM and 100 μM, respectively. The control sample contained the cells and a standard medium without any active agents. The MDA-MB-231 cell migration was assessed by monolayer gap closure migration assay, embedded by free ImageJ software (version 1.50i, National Institute of Health, Bethesda, MD, USA), with a wound healing tool macro (Montpellier RIO Imaging, CNRS, Montpellier, France). The area of the initial wound was measured, followed by gap area measurements after 8 h, 16 h, and 24 h. The migration factor was presented as the gap area value over the initial scratch area.

### 2.7. Statistical Analysis

All results are expressed as means ± SD and were obtained from three separate experiments and performed in quadruplicates (*n* = 12). The results were performed with independent sample *t*-tests. The experimental means were compared to the means of untreated cells harvested in a parallel manner. Differences between 24 h/48 h and control samples were tested for significance using the one- and multiple-way Friedman ANOVA test. A *p*-value less than 0.05 was considered statistically significant.

## 3. Results

**Figure 1:**
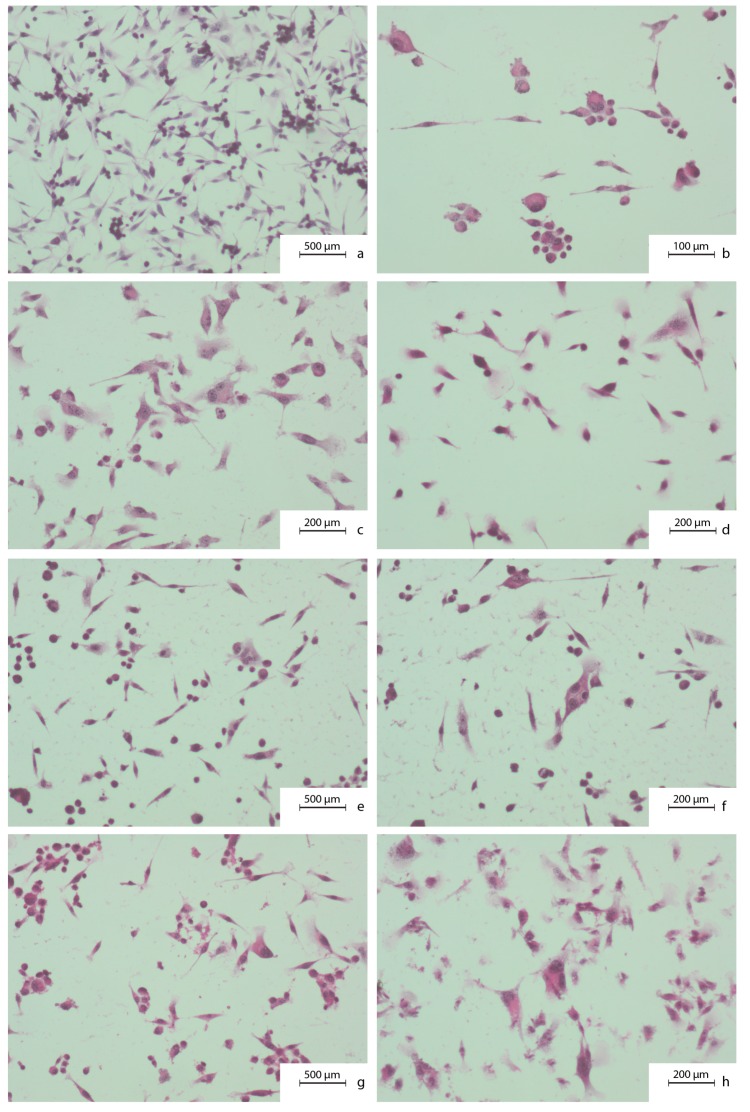
Cytomorphological view of MDA-MB-231 breast cancer cells without any treatment (**a**–**d**) as well as after 24 h of caffeic acid (CA) (**e**,**f**) and 24 h of caffeic acid phenethyl ester (CAPE) treatment (**g**,**h**), both with a 50 µM dose. To prepare the samples a hematoxylin and eosin staining was used. Exposition: optical magnification ×100 (**a**,**e**,**g**), ×400 (**c**,**d**,**f**,**h**), ×600 (**b**). Main features: (**a**) phenotypically as spindle-shaped cells (caudate, tadpole), hyperchromasia; (**b**) irregular nuclear shapes, small clusters of cells around the large ones; (**c**) large nuclei with irregular shape and several nucleoli in the nucleus; (**d**) pleomorphism of size, shape, and coloration of nuclei and whole cells; (**e**) karyopyknosis; (**f**) lower cell-cell contact; (**g**) karyopyknosis, cytoplasm density; (**h**) cytoplasm density and a shape change, cytoplasm fragmentation.

To obtain the quantitative assessment of breast cancer cells’ viability, the XTT-NR-SRB (Tetrazolium hydroxide-Neutral Red-Sulforhodamine B) assay was used. IN a parallel fashion, the effects of selected times/concentrations of CA and CAPE on breast cancer cell motility and migration were evaluated. Figure 1 shows MDA-MB-231 carcinoma cells’ morphology features as well as the impact of CA and CAPE on these cells. Examined cells were as phenotypical as the spindle shaped cells, with a visible hyperchromasia. Cell nuclei shapes were irregular. Small cells clustered around the large ones. The large, irregular nuclei contained several nucleoli in the nucleus. A pleomorphism of size and shape, as well as a coloration of nuclei, were visible. In the optical microscope, morphological characteristics of the apoptotic cells were visible, after the CA and CAPE treatment. Namely, we observed a cytoplasm density and changes in nuclear chromatin. The cytoplasmic shapes were changed. A fragmentation of a cytoplasm was visible. The cells were separated from each other.

**Figure 2:**
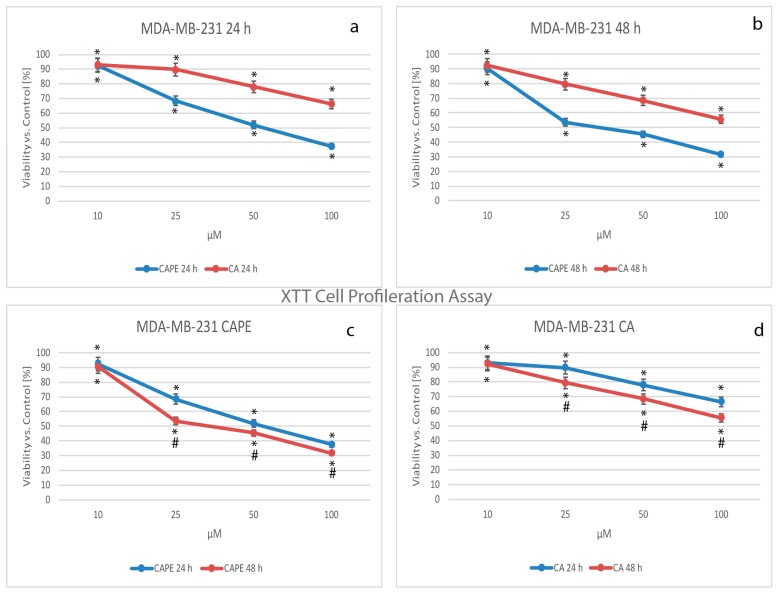
Cytotoxic effects of caffeic acid phenethyl ester (CAPE) and caffeic acid (CA) were both tested using concentrations of from 10 to 100 µM with 24 h and 48 h incubation times on the breast cancer cell line MDA-MB-231 using XTT (2,3-bis[2methoxy-4-nitro-5-sulfopheny]-2H-tetrazolium-5-carboxyanilide inner salt) Cell Proliferation Assay. Both polyphenols caused visible dose-dependent effects. Stronger activity was observed for CAPE than CA starting with a dose of 25 µM of each agent following 24 h (**a**) and 48 h (**b**) incubation times. A CAPE treatment of 48 h gave slightly stronger cytotoxic effect compared to 24 h (except a 10 µM dose) and was exclusively stronger for the 25 µM dose (**c**); however, succeeding dose increases of CAPE (50 and 100 µM) didn’t yield symptomatic difference in viability, with both times reaching a low level. The experiment times (**c**,**d**) had only a small impact on cytotoxic activity. The results were presented as a mean and standard deviation of three independent experiments, with 12 wells each (*p* < 0.05; Friedman ANOVA test; *—significant difference vs. control, #—significant difference 48 h vs. 24 h).

For years, tetrazolium salts have been widely used as detection reagents in histochemical localization studies and cell biology assays. Like the MTT assay (reducing tetrazolium dye: 3-(4,5-dimethylthiazol-2-yl)-2,5-diphenyltetrazolium bromide to formazan), XTT measures cell viability based on the activity of enzymes in mitochondria of live cells, which reduces XTT and becomes inactive shortly after cell death. The data obtained in the experiment were normalized and presented as the percentage of control values (Figure 2).

When CA was used for treatment of MDA-MB-231, cell viability decreased as the dose increased, dropping from 93.1% for a dose of 10 µM, 89.8% for 25 µM, 77.9% for 50 µM, and a value of 66.4% was reached with a dose of 100 µM after 24 h (Figure 2a,d). Simultaneously, when CAPE activity was compared to that of CA against MDA-MB-231 cells (Figure 2a,d), CAPE cell viability values for a dose of 10 µM were similar to CA (at 24 h, CA was 93.1%, while CAPE was 92.4%; after 48 h, CA was 92.4% and CAPE was 90.4%). The smallest doses of these two polyphenols had a similar cytotoxic effect on the examined cells. The effect increased in a dose-dependent manner for both agents. For CAPE, the values reached 68.4%, 51.9%, and 37.5% for respective doses of 25, 50, and 100 µM (Figure 2a,c), meaning that a stronger cytotoxic effect was achieved with CAPE at 24 h. After 48 h of incubation (Figure 2b), for both CAPE and CA, cell viability showed a dose-dependent effect and the values were as follows: for a 10 µM dose CAPE was 90.4% and CA 92.4%, for a dose of 25 µM CAPE was 53.5% and CA 79.5%, for 50 µM CAPE was 45.3% and CA 68.5%, and finally, for 100 µM CAPE was 31.6% and CA 55.5%.

Comparing CAPE activity to that of CA, viability was again lower for CAPE at the same dosage after 48 h. This showed a dependent trend for the dose and time domain (smaller impact) for both examined substances (Figure 2c,d). 

**Figure 3:**
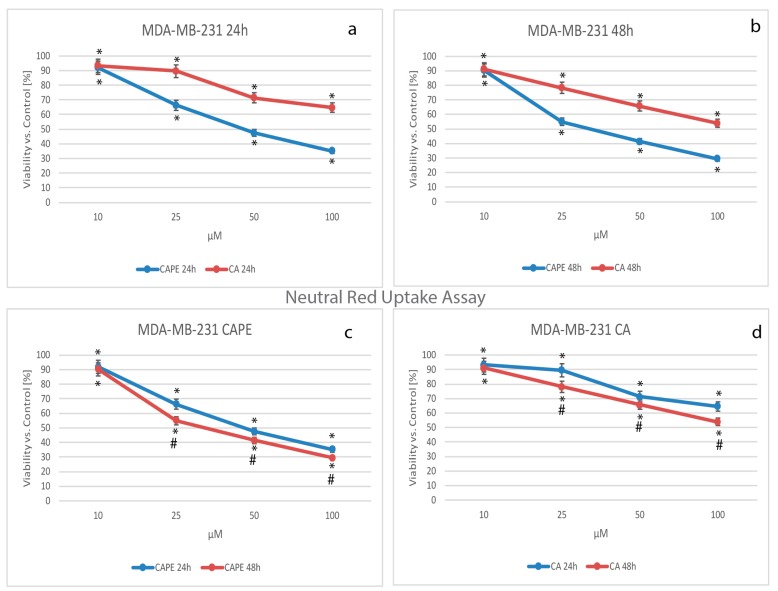
Cytotoxic effects of caffeic acid phenethyl ester (CAPE) and caffeic acid (CA) were tested using concentrations of from 10 to 100 µM with 24 h and 48 h incubation times on the breast cancer cell line MDA-MB-231 using neutral red (NR) Assay. Both polyphenols caused visible dose-dependent effects. A higher mortality factor was observed with CAPE than CA, starting from a dose of 25 µM of the tested compounds (**a**,**b**) for both 24 h and 48 h periods. In (**c**), using a dose of 10 µM of CAPE, the 48 h experiment did not produce any significant cytotoxic effects when compared to 24 h; nevertheless, a conspicuously stronger effect for 25 µM was observed. The succeeding dosage increases of CAPE (50 and 100 µM) displayed only a slight difference in viability factor, with both reaching a very low level. The cytotoxic activity of both substances showed no spectacular difference over time (**c**,**d**). The results were presented as mean and standard deviation of three independent experiments, with 12 wells each (*p* < 0.05; Friedman ANOVA test; *—significant difference vs. control, #—significant difference 48 h vs. 24 h).

The key component of the next viability test performed was the vital dye, neutral red (NR). Viable cells take up the dye by active transport and incorporate the dye into lysosomes, whereas non-viable cells do not take up the dye. The data obtained in the experiment were normalized and presented as % of viability over controls (Figure 3).

Using CA against MDA-MB-231 cells, the cell mortality increased in a dose-dependent manner. The viability values dropped from 93.26% for a dose of 10 µM, to 89.56% for 25 µM, 71.39% for 50 µM, and 64.54% with a dose of 100 µM of CA after 24 h (Figure 3a,d). Comparing CAPE’s cytotoxic activity to that of CA against MDA-MB-231 cells (Figure 3a,b), cell viability values for a dose of 10 µM were similar: at 24 h CA was 93.26% and CAPE was 91.96%, while at 48 h CA was 91.08% and CAPE 90.36%. A dosage of 10 µM of both polyphenols had a similar cytotoxic effect on the examined cells (independent of time). Using CAPE against the examined cells, at 24 h the values reached 66.30%, 47.40%, and 35.12% for doses of 25, 50, and 100 µM, respectively (Figure 3a,c). The results sustain that CAPE achieved a stronger cytotoxic effect at 24 h.

For both substances, cell viability manifested a dose-dependent effect after 48 h of incubation (Figure 3b). The values were: at 10 µM CAPE was 90.36% and CA 91.08%, at 25 µM CAPE was 55.02% and CA 78.25%, for 50 µM CAPE was 41.38% and CA 65.80%, and finally, for 100 µM, CAPE was 29.46% and CA: 53.86%. Therefore, CAPE induces greater cell mortality than CA at the same dosage. Both CA and CAPE showed a dependent trend for the dose and time domain, but again a smaller impact (Figure 3c,d).

**Figure 4:**
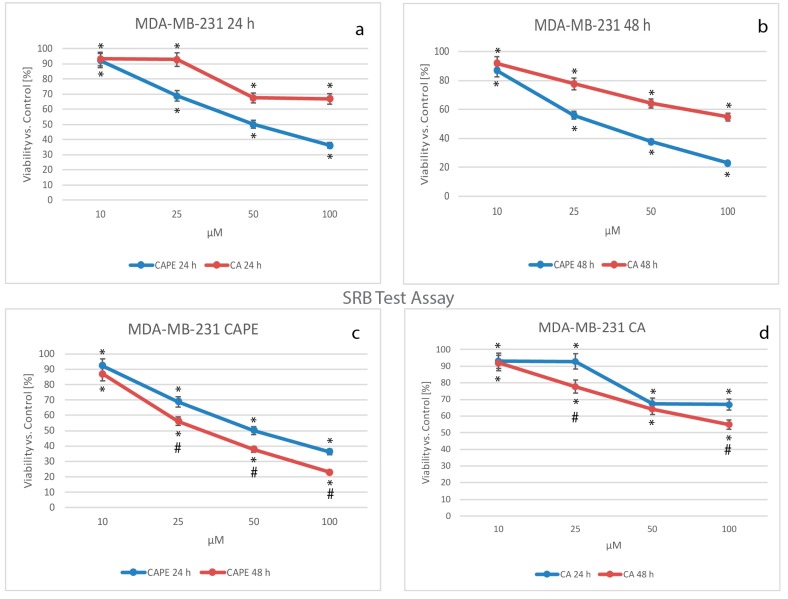
Viability results of the SRB (Sulforhodamine B) assay of caffeic acid phenethyl ester (CAPE) and caffeic acid (CA) at concentrations of from 10 to 100 µM for 24 h and 48 h incubation times on the breast cancer cell line MDA-MB-231. Like the XTT (2,3-bis[2methoxy-4-nitro-5-sulfopheny]-2H-tetrazolium-5-carboxyanilide inner salt) and NR (neutral red) tests, there was a visible dose-dependent effect for both polyphenols. Interestingly, for the 24 h experiment, CA (**a**) expressed ‘two levels’—a first for 10 and 25 µM and a second (**d**), for 50 and 100 µM; this phenomenon could be explained by nonlinear absorbance; however, within a 48 h experiment it does not exist. Greater cancer cell mortality using CAPE rather than CA started again (just as with XTT and NR) from a dose of 25 µM of each tested compound for both 24 h (**a**) and 48 h (**b**) incubation times. After 48 h (**c**), CAPE treatment showed a stronger cytotoxic effect in comparison to the 24 h period (except the 10 µM dose). The experiment time had only slight impact on the cytotoxic activity of the tested compounds (**c**,**d**), which wasn’t in opposition to the XTT and NR test assay. The results were presented as mean and standard deviation of three independent experiments 12 wells each (*p* < 0.05; Friedman ANOVA test; *—significant difference vs. control, #—significant difference 48 h vs. 24 h).

The key component of the last cytotoxicity test performed was the dye, Sulforhodamine B (Acid Red 52). An increase or decrease in the number of cells causes an associated change in the amount of dye incorporated by the cells in the culture. This indicates the specific degree of cytotoxicity caused by the test material. Data received during the experiment were normalized and presented as % of viability over controls (Figure 4).

Testing CA against MDA-MB-231 cells, cell viability declined in a dose-dependent manner, falling after 24 h from 93.13% for a dose of 10 µM, to 92.78% for 25 µM, 67.46% for 50 µM, and to a value of 66.89% using a dose of 100 µM CA (Figure 4a,d). When CAPE cytotoxic activity was compared to CA against MDA-MB-231 cells (Figure 4a,b), cell viability values for a dose of 10 µM were again close to those of CA. At 24 h, CA was 93.19% and CAPE 92.21%, and at 48 h, CA was 91.99% while CAPE was 86.90% (where a slight difference was finally observed). At a dosage of 10 µM, both polyphenols had a similar cytotoxic effect on the examined cells, which was also observed in viability tests performed earlier for this study. The viability was dependent for the dose and time domain. For CAPE at 24 h, the values reached 68.85%, 50.05%, and 36.13% for doses of 25, 50, and 100 µM, respectively (Figure 4a,c). Again, CAPE’s stronger cytotoxic effect than CA’s was confirmed after 24 h.

After 48 h of incubation (Figure 4b) for both CAPE and CA, cell viability revealed a dose-dependent effect, with values as follows: for 10 µM CAPE was 86.90% and CA 91.99%, for 25 µM CAPE was 56.08% and CA 77.69%, for 50 µM CAPE was 37.80% and CA 64.22%, and finally, 100 µM CAPE reached 22.98% and CA 54.88%. Comparing CAPE to CA after 48 h, cell mortality was again higher for CAPE than CA at the same dosage. Dependent trends in the dose and time domain (greater impact than by XTT and NR) for both CA and CAPE were confirmed (Figure 4c,d).

For both substances (CA and CAPE) used for the MDA-MB-231 breast cancer line, the half maximal inhibitory concentration (IC_50_) was calculated by all three methods during the experiment. It is significant that the 50%-mortality of breast cancer cells (MDA-MB-231) were ca. twice as low for CAPE than CA for all methods. This showed that CAPE has a stronger cytotoxic effect on MDA-MB-231 cells than CA during 24 h and 48 h experiments. The IC_50_ results are shown in Table 1.

Considering the cytotoxic effect of CA and CAPE (measured in this study by three methods), we clearly see that these two substances are active against MDA-MB-231 breast cancer cells, with CAPE displaying IC_50_ values more than twice as low as CA.

The next stage was an analysis of CA and CAPE’s influence on migration of MDA-MB-231 cells. This was measured by wound healing, which is the complex, dynamic process of movement and replacement of missing cells. Observation of live cells’ motility is an effective method to measure the rate of migration into the space created by the original wound. The desired situation is when the wound closes as little as possible, so the gap area value over the area of the original wound remains as great as possible, preferably for a prolonged period; this means the examined agent inhibits the migration of the carcinoma cells.

**Figure 5:**
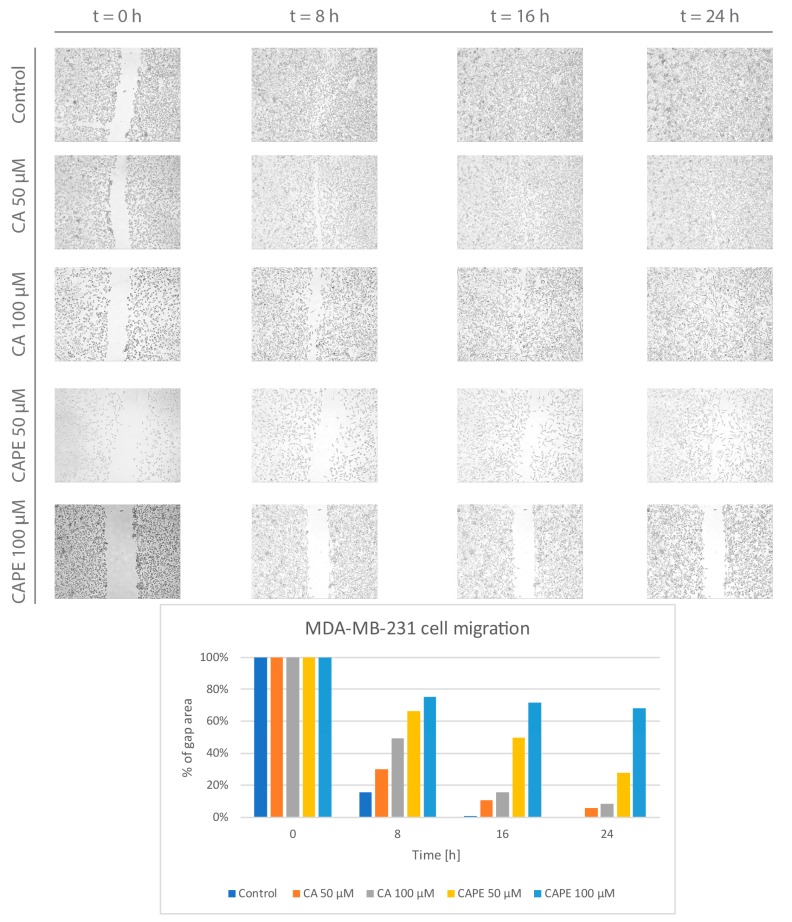
Caffeic acid (CA) and caffeic acid phenethyl ester (CAPE) at concentrations of 50 µM and 100 µM promote an inhibitory migration effect on MDA-MB-231 cells. There was a visible dose-dependent effect. The gap did not reach full closure for either agent. Comparison of these two substances shows CAPE has a greater influence on cell migration inhibition in MDA-MB-231 than CA. CAPE treatment with a dose of 100 µM demonstrated that the wound area basically remained unchanged over time. CAPE created a 'barrier’ that was practically impassable and impenetrable by the MDA-MB-231 cells. The cell migration factor was performed by monolayer gap closure migration assay and embedded by free ImageJ software. The results are presented as the gap area in relation to the area value of the initial scratch, after 8 h, 16 h, and 24 h of observation.

The results of the wound healing assay are presented in Figure 5. In the control group, cell migration was very dynamic, achieving a value of 16% after only 8 h. As seen, the wound’s closure was practically complete, reaching a rate value of 1% after 16 h. There was no evidence of the wound after 24 h. Using a 50 µM dose of CA, the motility of the MDA-MB-231 cells was inhibited. The rate increased to 30% after 8 h, 11% after 16 h and 6% after 24 h. The wound closure was not complete following the CA treatment. Increasing the CA dose to 100 µM resulted in better closure rates and therefore promoted migration inhibition of the MDA-MB-231 cells. The wound area value was 49% in relation to the original scratch after 8 h. A value of 16% was achieved after 16 h, and, finally, 9% after 24 h. Inhibition of the cell migration showed a dose-dependent trend.

Using CAPE for wound healing resulted in deeper inhibition of cell migration when compared to CA. For a CAPE dose of 50 µM, the gap area factor was 66% for 8 h. The gap remained at 50% at 16 h and achieved a value of 28% after 24 h. Increasing the CAPE dose to 100 µM displayed better results, as was expected. CAPE stopped the MDA-MB-231 cells from migrating at 75%, after 8 h. The size of the gap remained stable, with a value of 72% after 16 h, to reach a minimal value of 68% after 24 h. CA and CAPE both inhibited migration of MDA-MB-231 cells in a dose-dependent manner. The CAPE treatment displayed better results, particularly for the 100 µM dose, where the motility of tested breast carcinoma cells was practically halted.

## 4. Discussion

Research targeting finding new anticancer therapies is prompted by cancers’ high mortality rate. Bioactive compounds have taken their place in the research arena as new, effective medicines [59,60]. Phytochemicals such as flavonoids, polyphenols, and phenolic acids are of great interest to scientists, due to their specific, active, anticancer effect on cancer cells [61,62].

Among patients diagnosed with breast cancer, complementary and targeted therapies using alternative natural substances are often employed. Between 63% to 80% of all breast cancer patients use at least one type of alternative medicine, while herbal or vitamin therapies are used by some 25% to 63% of the same patient group [63,64,65,66,67].

Simonetti et al. showed that CA is bioavailable and it may be correlated with the antioxidant potential of plasma, by intake of red wine [68].

The presence of the estrogen receptor is one of the priority classification factors of breast cancer cells [18,69]. For breast cancer, proliferation and survival of the cells is dependent on estrogen receptor signaling [22,70].

TNBC can be perfectly modeled using the MDA-MB-231 line because there are no estrogen receptors α and expression of estrogen receptor β is minimal [71,72]. It was initially classified as a basal line of breast cancer cells because of the lack of ER and PR expression, as well as HER2 amplification. At present, it is considered to belong to the claudin-low molecular subtype because the line displays a down-regulation of claudin-3 and claudinin-4, as well as low expression of the Ki-67 proliferative marker and an enrichment of markers associated with an epithelial-mesenchymal transition and an expression of traits associated with breast cancer stem cells (CSC), such as CD44+/CD24−/low phenotype. The cells of this line are distinguished by invasive phenotype [72]. In a bone metastasis researches, the MDA-MD-231 cell line was widely used [73]. Also, the MDA-MB-231 cell subclones have been isolated. They displayed easy bone, brain, and lung metastases, after intraventricular injection into a mice organism. It allowed for the identification of genes and pathways that are potential mediators of metastasis to the specific sites when using this cell line [69,74,75,76,77].

For this research, we used MDA-MB-231 cell line as a model of TNBC. Considering the above, this comparative study of CA and CAPE substances, which occur naturally in propolis, shows much promise for breast cancer research.

The estrogenic effect of CAPE was not fully investigated, however; its ability to bind estrogen receptors has been previously shown. CAPE modulates the estrogen receptor selectively and it is more likely related to the estrogen receptor β than α [22,69]. This may indicate that the estrogen-related compounds act better on estrogen-positive neoplastic cells [25,78].

Khoram et al. [79] showed that CAPE stimulated radiosensitivity in breast cancer cells. Clonogenicity was inhibited and radiation-induced DNA damage was maintained in two cell lines, particularly in T47D cells.

Chen et al. [28] and Lin et al. [80] observed that CAPE’s anticancer activity was due to cell growth inhibition and a viability decline, both in a time and a dose-dependent manner. In another study, CAPE reduced the colony formation ability of PC-3 prostate cancer cells [26].

In an in vivo study, Wu et al. [27] showed CAPE’s ability to reduce the volume of breast cancer tumors, respectively, by 40% and 60% for MDA-231 and MCF-7 xenografts. Interestingly, the lower dose of CAPE (10 µM) was more effective to inhibit the growth of MDA-231 xenografts than 50 µM for the MCF-7 xenografts.

In earlier research, we compared the in vitro cytotoxic activity of ethanol extract of propolis and CAPE against two cell lines, MDA-MB-231 and Hs578T, using MTT and lactate dehydrogenase (LDH) assays. IC_50_ values obtained for CAPE (both assays) were definitely lower than for ethanol extract of propolis [39].

Watabe et al. showed that CAPE inhibits nuclear factor NFκB. They also examined CAPE to confirm that death-inducing receptors clustered. They found that Fas death-inducing receptors were aggregated through a Fas-L independent mechanism in the MCF-7 cells. Consequently, it was shown that CAPE induced apoptosis. The aggregation of death receptors was executed through two pathways; FADD/caspase-8 and JNK/p38 [38].

Beauregard et al. tested CAPE and its 18 derivatives against breast cancer MCF-7 cells. Induction of caspase 3/7 resulted in apoptosis in five of eighteen CAPE-derivatives, which was even better than CAPE alone. Inhibition of NFκB was similar for all tested analogs and CAPE itself. They found that activation of the p53 pathway was realized by all CAPE derivatives [81].

Rosendahl et al. [82] tested caffeine and CA against breast cancer cells MCF-7, T47D, and MDA-MB-231. Their results showed that CA inhibited the proliferation of breast cancer cells, reducing the growth of breast cancer cells through modulating ER and IGFIR levels, thereby influencing downstream effectors and cell-cycle progression, but better CA activity in the MCF-7 cells (estrogen-positive) was observed. Their results displayed that CA suppressed the proliferation of breast cancer cells. They also tested an influence of coffee intake on a breast cancer disease. A higher coffee intake was correlated with a smaller invasion of primary tumors. On the other hand, it was reported by Wu [23] that CAPE inhibits MCF-7 and MDA-231 cells growth. In both cell lines, CAPE induced apoptosis and cell cycle arrest, and inhibited NF-κB as well as down-regulated the mdr-1 gene. VEGF formation was also suppressed by MDA-MB-231 cells. We can assume that activity of CA is closely related to the expression of estrogen, while CAPE acts independently of estrogen.

Our results showed that CAPE has a better cytotoxic effect than CA, which is in line with other research. However, our comparison of these two agents is novel, as it uses a triple cytotoxic assay. 

Breast cancer metastasis is one of the primary reasons for its high mortality rate; therefore, migration and invasion research, as well as their mechanism, are part of the new era of breast cancer studies.

Wadhwa et al. used a free form of CAPE, as well as CAPE in a complex with gamma cyclodextrin (γCD) (equivalent doses), for cell viability studies of breast cancer lines MCF-7 and MDA-MB-231. They showed that CAPE displayed short-term toxicity, while CAPE-γCD complex caused permanent growth inhibition or apoptosis, which suggested that CAPE-γCD complex was characterized by a stronger effect. They also found that CAPE causes upregulation of p53 function by targeting mortalin-p53-interaction. The scratch and invasion studies on MCF-7 and MDA-MB-231 cells and their metastatic samples have shown that both CAPE-γCD complex and CAPE alone exhibit anti-migration activity [83].

Bonuccelli et al. showed that CAPE treatment significantly reduced wound closure (about 70% vs control) on breast cancer MCF-7 cells. In the 24 h period, CAPE acted as a natural mitochondrial OXPHOS inhibitor, which preventively targeted stem-like cancer cells. They also suggested that CAPE blocks formation of the mammosphere [84].

Recent research is being conducted to find a mechanism of the breast cancer cells migration.

Interesting results were proposed by Buchegger et al. [85]. They suggested potential mechanism of migration of MDA-MB-231 cells. They expressed Reprimo (RPRM) ectopically in MDA-MB-231 cells. RPRM is located at 2q23 and encodes a highly glycosylated protein that shows four bands (16, 21, 23, and 40 kDa) found predominantly in the cytoplasm. They found that RPRM overexpression suppressed migration and invasion of MDA-MB-231 cells. Another study on a mechanism of the migration was done by Bhat et al. [86]. Growth-regulated oncogene α (GROα) is a chemokine that plays a role not only in inflammation, but also in tumorigenesis. They found that MDA-MB-231 cells without GROα exhibited a significant migration decrease and invasion properties reduction. Liu et al. [87] showed that CD74 is involved in breast carcinoma metastasis. CD74 protein is the invariant chain of major histocompatibility complex (MHC) class II. Their results showed that this factor was highly expressed in MDA-MB-231 cells; furthermore, a downregulation of CD74 inhibited both migration and invasion of MDA-MB-231 cells. Wang et al. [88] reported that TBC1D3 oncogene promotes the migration of breast cancer cells, and its interaction with calmodulin enhances the effects of TBC1D3.

CAPE is known as a specific inhibitor of activation of nuclear transcription factor NF-κB in breast cancer cells [38,89]. Also, Wang et al. [90] found that an activation of NF-κB is required for the cell migration and TBC1D3-induced expression of OLR1, an oxidized low-density lipoprotein receptor 1, also known as lectin-like oxidized low-density lipoprotein (oxLDL) receptor-1. Our results showed a motility inhibition of human breast cancer cells by CAPE. It appears that CAPE addition inhibited the ability of the oncogene TBC1D3 to stimulate OLR1 expression in MDA-MB-231 cells. The tumor cells migration might be induced by TBC1D3, therefore an inhibition of NF-κB could result in the migration suppression thanks to CAPE addition.

CAPE influence on the migration of lung cancer A549 cells was tested by Shigeoka et al. They found that CAPE suppressed the motility promoted by TGF-beta-induced Akt phosphorylation [89].

Today, natural resources are being used more often. Also, a synthesis of analogues from natural remedies is proving to be an interesting source of substances that exhibit favorable activity for breast cancer treatment [90,91].

In earlier studies, we investigated the effect of CA on wound scratch on human squamous cell carcinoma cell line SCC-25. For ethanol treatment, approximately 5% of the wound was visible, while there was no closure if CA or a CA/ethanol mixture were used after 12 h. Total or nearly complete closure occurred after a treatment with 50 and 100 mmol/L of ethanol after 30 h, while with CA, a dose of 50 μM significantly inhibited migration of the cancer cells, leaving from 30% to 40% of wound closure after 30 h. The biggest gap (approx. 80%) was observed for pure CA treatment with a dose of 50 μM after 48 h [34].

We also compared the cytotoxic properties (by MTT) of CA and CAPE apoptosis induction and cell cycle arrest capabilities against MDA-MB-231 cells and found better activity of CAPE, with the same dosage and time of experiment [92].

Our novel comparative study confirms that CA and CAPE suspended migration rate of breast cancer MDA-MB-231 cells; however, much better results were obtained by the CAPE treatment. 

## 5. Conclusions

In this limited in vitro study, we showed a comparison of CA and CAPE, two bioactive substances isolated from bee propolis. An XTT-NR-SRB assay and migration evaluation by wound healing assay were performed. We strongly believe, based on our results and other reports, that CA and CAPE can be used for chemoprevention. Nevertheless, more advanced studies are needed, particularly clinical trials. The mechanism of CA and CAPE’s anticancer activity is becoming more well understood and documented; however, it remains a field in need of further investigation. Hopefully, this new approach of testing natural agents for breast cancer research will carefully explore the anti-cancer properties of all polyphenols. Our comparison of the effect of CA and CAPE on MDA-MB-231 cells clearly showed better results for CAPE-producing anticancer properties using the same dosages and experiment times.

## Figures and Tables

**Table 1:** IC_50_ (µM) values of caffeic acid (CA) and caffeic acid phenethyl ester (CAPE) in relation to breast cancer MDA-MB-231 for 24 h and 48 h, using different methods (XTT, NR, SRB, respectively: 2,3-bis[2methoxy-4-nitro-5-sulfopheny]-2H-tetrazolium-5-carboxyanilide inner salt, neutral red, Sulforhodamine B). All data demonstrated that lower doses of CAPE (ca. twice as low as CA) are needed to receive a similar mortality effect on MDA-MB-231 cells.

Method	Compound	Time of Incubation	Time of Incubation
		24 h	48 h
XTT	CA	150.94	108.42
	CAPE	68.82	55.79
NR	CA	135.85	103.23
	CAPE	64.04	53.25
SRB	CA	139.80	103.98
	CAPE	66.86	47.73
